# Mitral Valve-in-Ring Approach for High-Risk Pannus-Related Mitral Stenosis

**DOI:** 10.7759/cureus.94204

**Published:** 2025-10-09

**Authors:** Hassan O Yera, Ziyad Azam, Najeeb M Azam

**Affiliations:** 1 Internal Medicine, The Shrewsbury and Telford NHS Trust, Telford, GBR; 2 Internal Medicine, University Hospital North Midlands, Stafford, GBR; 3 Cardiology, The Shrewsbury and Telford NHS Trust, Telford, GBR

**Keywords:** mitral regurgitation, mitral stenosis, mitral valve-in ring, pannus formation, transcatheter mitral valve-in-valve replacement

## Abstract

A male patient under follow-up for degenerative mitral regurgitation, treated with a Physio annuloplasty ring two decades earlier, developed progressive shortness of breath and fluid overload, corresponding to New York Heart Association (NYHA) class III/IV. A transoesophageal echocardiogram revealed significant mitral stenosis, with a mean gradient of 11.8 mmHg due to pannus formation around the annuloplasty ring. Surgical repair posed a mortality risk of 25%-35% because of the combined risks of redo surgery, reduced left ventricular systolic function and chronic kidney disease. A transcatheter mitral valve-in-ring (TMViR) procedure was the only feasible option. A 29 mm Sapien 3 valve (Edwards Lifesciences, Irvine, CA) was successfully implanted within the mitral ring. Four months following the procedure, the patient reported significant symptom relief and an improved quality of life (QOL), with a shift to NYHA class I/II. Follow-up echocardiography demonstrated a stable valve position, a mean gradient of 4 mmHg and mild mitral regurgitation. This case highlights TMViR as a viable option for high-risk patients with pannus-related mitral stenosis.

## Introduction

Surgical repair is a standard treatment for degenerative mitral regurgitation, commonly involving annuloplasty [1]. This technique has been refined over decades and is often preferred for preserving native valve function. Mitral stenosis following ring annuloplasty is less common but presents significant clinical challenges. It often results from structural changes to the annuloplasty ring or pannus formation, an excessive fibrotic overgrowth that can lead to left ventricular inflow obstruction [2,3]. Pannus formation is particularly associated with complete or undersized rings and is more likely to occur several years post surgery. The pathophysiological process involves chronic inflammatory responses to foreign material, leading to fibrosis and valve restriction. Historically, reoperation was the mainstay of treatment for pannus-related mitral stenosis. While surgical valve replacement is effective, it carries a high morbidity and mortality risk, particularly in elderly and frail patients with comorbidities [4]. Advances in imaging techniques such as four-dimensional (4D) echocardiography and computed tomography (CT) have improved the detection and evaluation of pannus, enabling better pre-procedural planning and alternative approaches. The transcatheter approach has revolutionised the management of valvular heart diseases, offering a minimally invasive option for high-risk patients [5]. Transcatheter mitral valve-in-ring (TMViR) procedures provide a promising alternative to surgery by treating haemodynamically significant stenosis without the need for open-heart surgery [4-6]. Careful patient selection and multidisciplinary collaboration are essential for achieving optimal outcomes with this innovative technique.

## Case presentation

**Figure 1:**
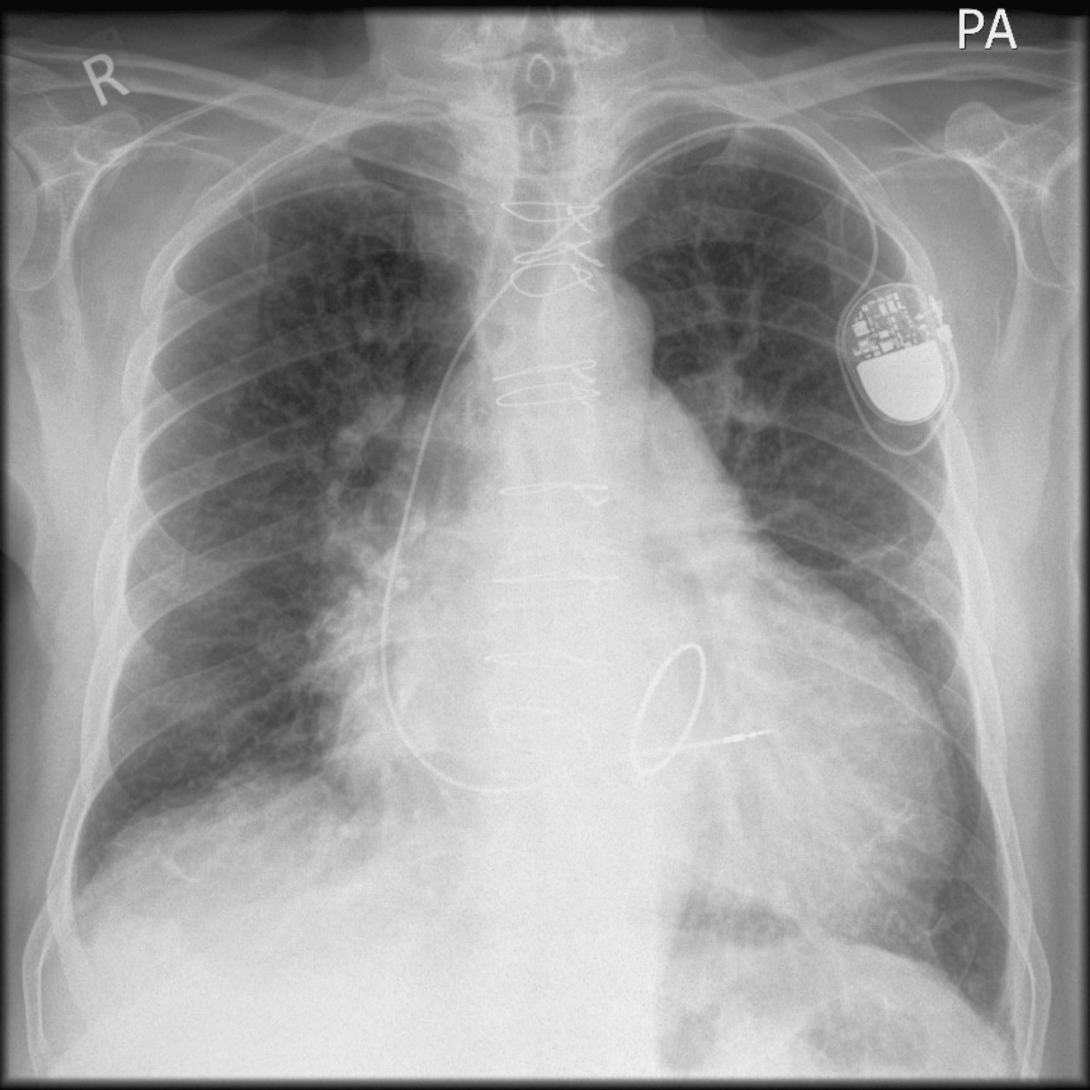
Chest X-ray displaying cardiomegaly and pulmonary congestion It also shows the mitral annuloplasty ring and sternotomy wires from previous surgical repair, along with a single-lead permanent pacemaker

A man in his 70s presented with worsening shortness of breath even on minimal exertion, limiting his ability to walk 100 yards, with New York Heart Association (NYHA) class III/IV. Examination revealed pedal oedema and lung congestion. Chest X-ray (Figure 1) confirmed cardiomegaly and pulmonary congestion. He was admitted for heart failure and successfully diuresed.

Two decades earlier, he had undergone mitral valve repair for degenerative mitral regurgitation, which involved decalcification, para-commissural edge-to-edge suture repair, central edge-to-edge double-orifice technique with a 38 mm Physio annuloplasty ring and radiofrequency ablation for atrial fibrillation. Despite these procedures, he needed a repeat catheter ablation and, later, a permanent pacemaker. His medical history was also notable for stable stage 3b chronic kidney disease.

Investigations

Baseline blood investigations are presented in Table 1. Transthoracic echocardiography (TTE) (Videos 1, 2) conducted during admission revealed moderate left ventricular systolic dysfunction, significant mitral stenosis with a mean gradient of 8 mmHg and mild-to-moderate eccentric regurgitation. Severe tricuspid regurgitation and a high probability of pulmonary hypertension were noted. Transoesophageal echocardiography (TOE) confirmed severe mitral stenosis (mitral valve area, 0.9 cm²; mean gradient, 11.8 mmHg) with mild mitral regurgitation. Four-dimensional TOE imaging showed notable tissue overgrowth (Video 3), consistent with pannus, leading to obstruction. Right ventricular systolic function was severely compromised, while left ventricular systolic function was mildly reduced. Renal function remained stable throughout.

**Table 1:** Baseline haematological and biochemical profile of the patient eGFR, estimated glomerular filtration rate; GT, glutamyl transferase

Test	Result	Unit	Reference range	Interpretation
Total white cell count	4.6	×10⁹/L	4.0-11.0	Normal
Red blood cell (RBC) count	3.33	×10¹²/L	4.50-6.50	Low
Haemoglobin	103	g/L	130-180	Low
Haematocrit	0.329	L/L	0.400-0.520	Low
Mean corpuscular volume (MCV)	98.8	fL	80.0-100.0	Normal
Mean corpuscular haemoglobin (MCH)	30.9	pg	27.0-32.0	Normal
Platelet count	119	×10⁹/L	150-450	Low
Neutrophil count	3.5	×10⁹/L	2.0-7.5	Normal
Lymphocyte count	0.4	×10⁹/L	1.5-4.5	Low
Monocyte count	0.5	×10⁹/L	0.2-0.8	Normal
Eosinophil count	0.1	×10⁹/L	0.0-0.4	Normal
Basophil count	0	×10⁹/L	0.0-0.1	Normal
Sodium	140	mmol/L	133-146	Normal
Potassium	4.5	mmol/L	3.5-5.3	Normal
Urea	23.1	mmol/L	2.5-7.8	High
Creatinine	174	µmol/L	60-110	High
eGFR (1.73 m²)	32	mL/minute	≥60	Low
Albumin	44	g/L	35-50	Normal
Alkaline phosphatase (ALP)	105	U/L	40-130	Normal
Alanine aminotransferase (ALT)	13	U/L	0-45	Normal
Bilirubin	13	µmol/L	0-21	Normal
Gamma-GT (GGT)	73	U/L	0-75	Normal

**Video 1:** Parasternal long-axis view with reduced flow across the stenotic mitral valve pre-procedure

**Video 2:** Zoomed-in apical four-chamber view of the mitral valve pre-procedure

**Video 3:** 4D transoesophageal echocardiogram of the mitral valve showing mitral ring with pannus tissue 4D: four-dimensional

Treatment

The cardiothoracic multidisciplinary team (MDT) determined that surgical intervention carried an unacceptably high mortality risk (25%-35%) according to the Society of Thoracic Surgeons (STS) score. As a result, the only viable alternative was a transcatheter mitral valve-in-ring (MViR) procedure. The patient was informed of the associated risks and benefits and provided informed consent. A pre-procedural CT scan was performed to guide vascular access and valve sizing.

Under general anaesthesia and with transoesophageal echocardiography guidance, a trans-septal MViR procedure was performed via the right femoral vein. A 29 mm Sapien 3 valve (Edwards Lifesciences, Irvine, CA) was successfully implanted into the existing 38 mm Physio II ring. The procedure was complicated by ventricular fibrillation during valve deployment; however, spontaneous circulation was restored with brief cardiopulmonary resuscitation and defibrillation. Post-procedure echocardiography confirmed the resolution of mitral stenosis, only mild residual mitral regurgitation and a well-seated valve. The patient required a short period of postoperative intensive care monitoring before step-down to the cardiology ward in stable condition.

Outcome and follow-up

At the four-month follow-up, the patient reported significant improvement in symptoms and quality of life (QOL), classified as NYHA class I/II. Transthoracic echocardiography (TTE, Video 4) showed a non-dilated left ventricle (ejection fraction {EF}: 40%), mild mitral regurgitation, a mean gradient of 4 mmHg and moderate (previously severe) tricuspid regurgitation. At the 12-month follow-up, he continued to report good QOL with no hospitalisations for heart failure.

**Video 4:** Apical four-chamber view with colour flow Doppler over the mitral area and the left atrium following the procedure

## Discussion

The European Society of Cardiology recommends percutaneous mitral commissurotomy or surgical repair for native mitral stenosis [1]. Although rare, pannus formation following mitral ring annuloplasty is challenging to manage, especially in elderly patients at high surgical risk. While surgical mitral valve replacement is typically the treatment of choice, it is associated with significant morbidity and mortality in such cases [7,8].

We report on the use of the novel transcatheter mitral valve-in-ring (TMViR) approach to treat haemodynamically significant pannus-related mitral stenosis in an elderly patient who was at very high surgical risk, two decades after ring annuloplasty for mitral regurgitation due to degenerative posterior mitral valve leaflet (PMVL) pathology.

TMViR has emerged as a promising alternative for patients with failed annuloplasty rings, offering a less invasive approach with acceptable short-term outcomes [4-6]. Although primarily used for recurrent mitral regurgitation, its application in pannus-related mitral stenosis remains underreported. This case highlights the efficacy of TMViR in restoring valve function and improving the quality of life in high-risk patients.

Recent advancements in TMViR technology have further improved patient outcomes. Innovations such as enhanced valve design, CT-guided procedural planning and refined balloon-sizing techniques have minimised complications and optimised valve seating [5]. Short-term results for TMViR procedures demonstrate mortality rates between 8.1% and 11.5%, with durability and functionality remaining satisfactory in most cases [4].

Patient selection remains crucial for procedural success. It is essential to identify individuals at high surgical risk who also have anatomically favourable conditions for TMViR. Multidisciplinary evaluation and multimodality imaging are vital in guiding these decisions, as demonstrated in this case.

Common complications of TMViR include left ventricular outflow tract (LVOT) obstruction, paravalvular leaks and arrhythmias [6]. In our case, ventricular fibrillation during valve deployment was effectively managed without long-term sequelae, underscoring the importance of immediate procedural support.

Although LVOT obstruction remains one of the most feared complications of TMViR, several preventive strategies have been developed. Among these, the laceration of the anterior mitral leaflet to prevent outflow obstruction (LAMPOON) procedure has shown promise in reducing the risk of LVOT obstruction in selected patients undergoing transcatheter mitral valve replacement. This electrosurgical technique involves the intentional midline laceration of the anterior mitral leaflet to preserve LVOT patency following valve deployment [9]. Early clinical studies have demonstrated its feasibility and safety in high-risk cohorts, with significant reductions in obstruction-related complications [10]. However, given the procedural complexity and the need for specific anatomical considerations, LAMPOON was not utilised in our case, as pre-procedural imaging did not predict a high risk of LVOT obstruction.

This report aligns with broader trends in TMViR outcomes for high-risk patients, reinforcing its potential to reduce surgical risk while delivering excellent functional results.

## Conclusions

TMViR is an innovative and effective treatment for haemodynamically significant pannus-related mitral stenosis in high-risk patients. Multidisciplinary teamwork is vital for optimising personalised patient care. Recent technological advances have improved TMViR outcomes, reducing procedural risks and boosting success rates. Thorough imaging and careful patient selection are crucial for achieving the best results in complex cardiac cases.

## Appendices

Patient's perspective

My mitral valve problem began 20 years ago when I had a repair that was quite successful. However, about two years ago, I started noticing deterioration as I began experiencing breathing difficulties during any strenuous activity, to the point where even walking a very short distance or getting dressed in the morning became a challenge, and many of my previous activities were restricted. It also began to strain my relationship with my partner, as she was very active. Having been under the care of a cardiologist for some time, to cut a long story short, his persistence secured me an appointment with his colleagues, which led to a procedure not often performed to correct my issue. I was subsequently admitted, and the procedure was successfully completed. To sum up that success, I find it hard to describe how I feel now, being able to get out of my car and walk across; even thinking about it is like 'magic!' I am deeply grateful to my consultants and their teams for giving me back my life, a second chance, if you like. Thanks, everyone! Moreover, I now enjoy a quality of life I never thought possible again; my thoughts before the procedure were, if this is as good as it gets, then 'take me out of here!'
